# A Longitudinal Study on Maternal Depressive Symptoms During the COVID-19 Pandemic: The Role of Strict Lockdown Measures and Social Support

**DOI:** 10.3389/ijph.2022.1604608

**Published:** 2022-03-14

**Authors:** Joana Fernandes, Inês Tavares, Pedro Bem-Haja, Tânia Barros, Mariana L. Carrito

**Affiliations:** ^1^ Faculty of Psychology and Education Sciences, University of Porto, Porto, Portugal; ^2^ Department of Education and Psychology, University of Aveiro, Aveiro, Portugal; ^3^ Centro Materno-Infantil do Norte, Porto, Portugal

**Keywords:** pregnancy, longitudinal, COVID-19, depression, postpartum

## Abstract

**Objectives:** This study examined the trajectory of perinatal depressive symptoms in Portuguese women during the COVID-19 pandemic and the role of individual, relational, and contextual risk and protective factors.

**Methods:** This 3-wave longitudinal study followed 290 pregnant women from the third trimester of pregnancy until 6-months postpartum. Women self-reported on depressive symptoms, psychological (anxiety, perceived stress, mindfulness), relational (perceived social support, dyadic adjustment, sexual wellbeing), and contextual (lockdown status) factors.

**Results:** Women who were under strict lockdown presented significantly higher scores of depressive symptoms at baseline (by 1.38 EPDS points) than women who were not under strict lockdown measures. Mixed Growth Models showed that trajectories of depressive symptoms were explained by differences in women’s baseline depression. Differences in women’s depressive symptoms at baseline were mainly explained by higher anxiety and lower social support (22% and 24% for women under lockdown; 39% and 6% for women not on lockdown, respectively).

**Conclusion:** Preventative interventions targeted at pregnant women should aim to reduce anxiety and enhance women’s social support to prevent depression in pregnancy and postpartum during the COVID-19 pandemic.

## Introduction

During pregnancy and postpartum, changes to women’s physiological, psychological, and relational functioning can affect mothers’ mental health [1], with direct and indirect consequences to their physical health, the couple’s relationship, and their child [2–4]. A recent review found that heightened life stress and low social support are the most common risk factors for postpartum depression and that prenatal depression is the strongest predictor of postnatal depression [5]. Indeed, the transition to motherhood is typically stressful for mothers, with both external stressors and individual factors contributing to the potential development of depression [6]. According to the vulnerability-stress-adaptation framework—a theoretical framework that aims to explain the development of psychopathology in several settings—, both distal and proximal factors contribute to the onset and maintenance of a disorder. According to this model, relatively minor stressors may lead to mental health problems in a highly vulnerable person, whereas a major stressful event might cause a similar reaction in a person with low vulnerability [6]. This model enables us to understand how stressful events may increase the risk of developing maternal psychopathology.

In contrast to common life stressors, the COVID-19 pandemic is considered a major and large-scale stressor that can potentiate mental health difficulties during an already vulnerable period for the occurrence of depression among women [7, 8]. During the current pandemic, pregnant women dealt with diverse concerns such as fear of infection, worries over their health and of their baby [9, 10], and a range of uncertainties about the future (e.g., regarding the childbirth process [10, 11]). Many healthcare practices were changed in an attempt to reduce the risk of infection and transmission of the virus. Simultaneously, confinement and social distancing measures implemented to prevent virus transmission resulted in increased social isolation and perceived loneliness [9–11]. Together, these measures have already been recognised as leading to increased psychological distress and a higher risk of developing psychological problems [9].

In the case of depression, the priorly identified risk factors can be exacerbated during the current pandemic and, in turn, may increase the risk of new mothers experiencing depressive symptoms. A recent review [12] identified factors that increase the likelihood of developing maternal postpartum depression during the pandemic, including the presence of depressive symptoms during pregnancy, prenatal anxiety symptoms, low social support during pregnancy, exposure to traumatic events during or prior to pregnancy, and high stress levels. While some consensus exists about risk factors implicated in postpartum depression, the effects of the COVID-19 pandemic on the likelihood of developing depressive symptoms, and which groups of mothers are at higher risk, is not clear. In Italy, several COVID-19 related stressors showed a significant effect on maternal depression. More specifically, women who indicated being scared of a COVID-19 infection reported higher levels of depressive symptoms compared to women who did not identify those fears [13]. Similar findings reinforce that those who experienced greater pandemic-related fears also reported increased anxiety and are at higher risk of developing postpartum depression [14]. Indeed, high anxiety experienced during the pandemic seems to be an important risk factor for depressive symptoms [11]. Nevertheless, a study conducted with postpartum mothers showed that women who gave birth during the pandemic presented a lower risk of experiencing depressive symptoms immediately after childbirth than a control group of mothers who gave birth a few years prior to the pandemic [15]. Although several recent studies have already examined the negative effect of the COVID-19 pandemic on maternal psychological health [16–18], it cannot be inferred that the COVID-19 pandemic increases the risk for developing depressive symptoms, and not in the same degree for all women.

The transition to parenthood can also result in interpersonal and sexual changes for the mother and her partner, which have been linked to depressive symptoms [19, 20]. However, few studies have examined how these changes occur over time as new parents adjust to new parenthood while navigating a global crisis. A systematic review of the literature from 18 countries assessing women’s sexual health during the COVID-19 pandemic showed deteriorations on sexual desire, sexual frequency, sexual satisfaction, and relationship satisfaction [21]. In first-time mothers and fathers, COVID-19-related concerns and personal psychological difficulties caused by the pandemic were identified as precursors of dyadic and sexual alterations [10, 22, 23]. Still, current evidence is not clear to support that frequency of sexual concerns precede change in depressive symptoms across pregnancy and postpartum [20]. Although the COVID-19 pandemic has exacerbated the negative effects on women’s mental and sexual health, some protective factors might protect them against emotional distress. Findings show that mindfulness-based skills and social support are moderately to strongly beneficial to manage pregnancy- and postpartum-related changes, as well as for dealing with stressful situations such as the current global pandemic [24, 25].

Using a longitudinal approach, the present study aimed to investigate how the COVID-19 pandemic impacted maternal mental health, particularly regarding the development of depressive symptoms, and whether pandemic-related strict confinement measures increased the risk of developing depressive symptomatology for these women. We specifically aimed to: 1) examine changes in maternal depressive symptoms over time from 28 weeks pregnancy to 6-months postpartum; 2) explore whether the imposition of a national emergency state due to the COVID-19 pandemic was associated with an increased likelihood of experiencing depressive symptoms; 3) assess risk and protective factors for women’s depressive symptomatology during the COVID-19 pandemic; and 4) compare a predictive model of depressive symptomatology between pregnant women during the COVID-19 state of emergency with a control group of women who were pregnant but not experiencing strick lockdown measures.

## Methods

### Participants and Procedure

Between October 2019 and May 2021, a convenience sample of pregnant women was recruited at regularly scheduled clinical appointments at a large outpatient unit in Portugal and via online advertisement. Pregnant women over the age of 18 years who were healthy (i.e., no major physical or psychological pathology at entry), in a commited relationship with a partner, and expecting their first child were eligible to participate.

The sample consisted of 290 women, with an average age of 30.1 years (SD = 4.58, range = 19–42). Women were married (40%), dating (31%), or in a common law relationship (29%). Participants mostly reported a high education (62%) and had a monthly household income of 1,260–2,514 € (50%), which corresponds to a middle-class income. Across the pandemic, Portugal declared two periods of national state of emergency (18th March–2nd^,^ May 2020; 9th November 2020–30th April 2021) which included mandatory and strict physical isolation and social distancing (i.e., “stay at home”) measures. These measures were similar across both state of emergency periods and across the whole country. The implementation of a national emergency state occurred when 55 of these women were in the third trimester of pregnancy, followed by 69 women who were at 3-month postpartum, and 107 women who were at 6-month postpartum.

The recruitment process started in 2019 and continued during the COVID-19 pandemic, providing the opportunity to compare symptoms of perinatal depression before and during the COVID-19 pandemic. Participants completed an online self-report survey at three waves (T1: 28 pregnancy weeks, all sample; T2: 3-month postpartum, 242 women; T3: 6-month postpartum, 224 women; retention rate = 77%). Participation was voluntary; all participants provided informed consent before participating. Each participant was compensated with a 10 € gift card at every other time-point of the study. At the end of the study, women received information on relevant psychological resources during the COVID-19 pandemic. The study was approved by the Ethics Committee at the University of Porto and at the Centro Materno-Infantil do Norte.

### Measures

#### Sociodemographic Information

Participants reported on sociodemographics such as age, marital status, education, and annual household income. Date of participation was collected to estimate whether participants were responding under or before a COVID-19 national emergence state.

#### Edinburgh Postnatal Depression Scale

The EPDS is a widely-used 10-item self-report measure to screen depressive symptoms particularly at pregnancy and postpartum [26]. On a four-point Likert-type scale, participants reported on the frequency of experiencing symptoms of depression in the last week. Higher total scores indicate higher presence of depressive symptoms. The EDPS has been validated for use in Portuguese samples [27]. In this study, internal consistency of the EPDS scale was very good (ordinal *α* = 0.90).

#### Hospital Anxiety and Depression Scale

The HADS is a self-report questionnaire to measure anxiety and depression [28], comprising two 7-item subscales rated on a four-point Likert-type scale: anxiety (HADS-A) and depression (HADS-D). In the current study, only the HADS-A was used as a measure of anxiety symptoms. Higher scores indicate greater anxiety. The HADS has been translated to Portuguese and validated for use in Portuguese samples [29]. In this sample, the scale showed good internal consistency (ordinal *α* = 0.89).

#### Dyadic Adjustment Scale-Revised

Relationship adjustment was assessed with the well-validated 14-item DAS-R [30]. Using a seven-point Likert-type scale, individuals report on aspects of relational functioning and satisfaction; higher scores indicate greater marital satisfaction. The DAS-R has been validated for the Portuguese population [31] and, in this sample, showed good internal consistency (ordinal *α* = 0.88).

#### Perceived Stress Scale

The 14-item PSS was used to assess women’s perceived stress in the last month [32]. Items are rated on a five-point Likert-type scale; higher scores denote higher perceived stress. The Portuguese version of the PSS yielded good internal consistency [33]. In our study, the scale demonstrated very good internal consistency (ordinal *α* = 0.90).

#### Multidimensional Scale of Perceived Social Support

The MSPSS is a 12-item measure that assesses perceived social support [34]. On a seven-point Likert-type scale, participants rate the perception of social support received from three sources (family, friends, and significant other). Higher total scores indicate higher perceived social support. The Portuguese validation yielded good psychometric properties [35]. In this study, the scale showed very good internal consistency (ordinal *α* = 0.96).

#### Five Facet Mindfulness Questionnaire

The 39-item FFMQ was used to assess mindfulness [36]. Using a five-point Likert-type scale, women indicate the degree to which each item applies to them. The scale has been validated for the Portuguese population [37] and, in the current study, showed good internal consistency (ordinal *α* = 0.72).

#### Female Sexual Distress Scale

The well-validated FSDS is a 13-item questionnaire that assesses sexual distress during the previous month [38]. Using a five-point Likert-type scale, women rate how often their current sexual experiences have bothered them or caused them distress. Higher scores indicate higher sexual distress. In this study, the scale showed very good internal consistency (ordinal *α* = 0.95).

#### Female Sexual Function Index

The FSFI is a 19-item questionnaire that measures female sexual functioning [39]. On a five-point Likert-type scale, participants rate their sexual functioning in several dimensions (desire, arousal, lubrication, orgasm, and pain). Higher total and subscales scores indicate better sexual functioning. The Portuguese validation of the scale demonstrated good psychometric properties [40] and, in this study, the scale showed very good internal consistency (ordinal α = 0.98).

### Statistical Analysis

All analyses were performed using R. To assess the longitudinal pattern (fluctuation) of EPDS across the three study moments (T1, T2, T3), we employed Growth Mixed Models. Growth models were estimated within a Linear Mixed Model framework, fit by REML. T-tests calculations used Satterthwaite’s method, confidence levels were computed using bootstrapping with the percentile method [2.5%–97.5%]. A sequential 3-step method was used for Growth Models: I) Unconditional Means Model, II) Unconditional Growth Model, and III) Conditional Growth Model. First, the Unconditional Means Model was used as the baseline model, providing a grand average of the initial EPDS without additional variables. The unconditional means model also allowed us to verify whether women showed significant variability in EPDS scores. Second, the Unconditional Growth Model was estimated with the inclusion of the variable Moment of pregnancy/postpartum (T1, T2, or T3) in order to evaluate the existence of significant fluctuation in EPDS scores over time. Third, a Conditional Growth Model with the inclusion of state of emergency as a moment-variant covariate was estimated. The state of emergency variable translates the presence versus absence of state of emergency measures in a dichotomous manner (state of emergency = 1, no state of emergency = 0) and was created following the calendar of restrictions imposed by the Portuguese Government at the time of response for each participant at each moment.

Finally, to examine the influence of potential risk and protective factors for depressive symptoms, we conducted a Relative Importance Analysis using Tonidandel and Lebreton’s [41] method. This analysis aimed to evaluate the predictive ability of known risk and protective factors (i.e., anxiety, perceived stress, perceived social support, dyadic adjustment, mindfulness, sexual function, and sexual distress) on women’s depressive symptomatology, and to examine differences in the predictive ability of these variables in the EPDS scores depending on whether women were under the state of emergency measures or not.

## Results

Findings from all estimated Mixed Models are described below; see Table 1 for a full depiction of results.

**TABLE 1:** Longitudinal analysis using four growth models to assess the fluctuation of depressive symptoms across the three moments of the study (Porto, Portugal, 2021).

Predictors	Model I			Model II			Model III			Model IV		
	Unconditional means model			Unconditional growth model			Conditional growth model			Conditional growth model		
	Estimates	CI	*p*	Estimates	CI	*p*	Estimates	CI	*p*	Estimates	CI	*p*
Fixed effects												
(Intercept)	6.42	5.98–6.86	**<0.001**	6.37	5.91–6.89	**<0.001**	6.09	5.61–6.59	**<0.001**	0.52	0.19–0.87	**0.004**
Moment				0.05	−0.17–0.29	0.638	0.26	−0.06–0.57	0.091	0.10	−0.23–0.39	0.538
Emergency State [1]							1.38	0.68–2.07	**<0.001**	0.94	−0.31–1.41	**0.017**
Moment*Emergency State [1]							−0.82	−1.37–-0.30	**0.003**	−0.26	−0.64–0.25	0.261
EPDS T1										0.91	0.85–0.93	**<0.001**
EPDS T1*Emergency State [1]										−0.08	−0.16–0.00	**0.058**
Random effects												
σ^2^	6.24			6.19			6.09			3.10		
τ_00_	13.51_Part_			12.03_Part_			11.63_Part_			1.86_Part_		
τ_11_				0.06_Part.Moment_			0.08_Part.Moment_			2.64_Part.Moment_		
ρ_01_				1.00_Part_			1.00_Part_			−1.00 _Part_		
ICC	0.68			0.69			0.61			0.495		
N	290_Part_			290_Part_			290_Part_			290_Part_		
Observations	753			753			753			753		
*R* ^2^	0.684			0.689			0.692			0.860		
AIC	4,058.7			4,060.7			4,051.4			3,429.8		
BIC	4,073.6			4,088.4			4,089.4			3,471.5		
−2LL	−2,026.9			−2,026.1			−2,020.3			−1,705.9		
Deviance based on ML	4,052.7			4,048.7			4,036.4			3,411.8		
Model I vs. Model II	*χ* ^2^ = 4.0203, df = 3, *p* = 0.259281											
Model II vs. Model III				*χ* ^2^ = 13.307, df = 2, *p* = 0.001289**								
Model III vs. Model IV							*χ* ^2^ = 616.85, df = 1, *p <* 0.0001***					

Bolded values indicate significant effects.

The Unconditional Means Model (Model I) showed a Grand Average (6.42) significantly higher than 0, CI 95% [5.99, 6.87]. Additionally, statistically significant variation in the intercept across participants CI 95% [3.28, 4.05] was found, indicating variability in depression scores across participants. In fact, the ICC showed that 68% of the total variation in EPDS was attributable to differences among participants. Considering these results, the temporal change for EPDS scores for each individual was visually assessed (see Trelis plot in Supplementary Material S1). The model showed significant variability for both intercepts and slopes, with some participants increasing on EPDS scores and others decreasing over time.

The Unconditional Growth Model (Model II) showed a maintenance of the intercept effect, CI 95% [5.89, 6.81], with the mean value of EPDS at baseline being 6.37. In this model, no moment effect was noted, CI 95% [−0.17, 0.27], with an average growth of 0.05 points in EPDS score per moment. Regarding random effects, we found statistically significant variation across women at baseline, CI 95% [2.62, 9.73] and in the slope, CI 95% [0.06, 0.96]. Although most of the variance was explained by differences between individuals, a significant variance within individual random error was also found, CI 95% [2.25, 2.63]. This showed a significant amount of variation between the observations at different moments and the individual regression lines. Because of the two statistically significant random-effect variances (τ00 and τ11), we were able to further explore potential individual-related variables. As such, the time-variant individual-related variable state of emergency was introduced in the Conditional Growth Model (Model III).

**FIGURE 1:**
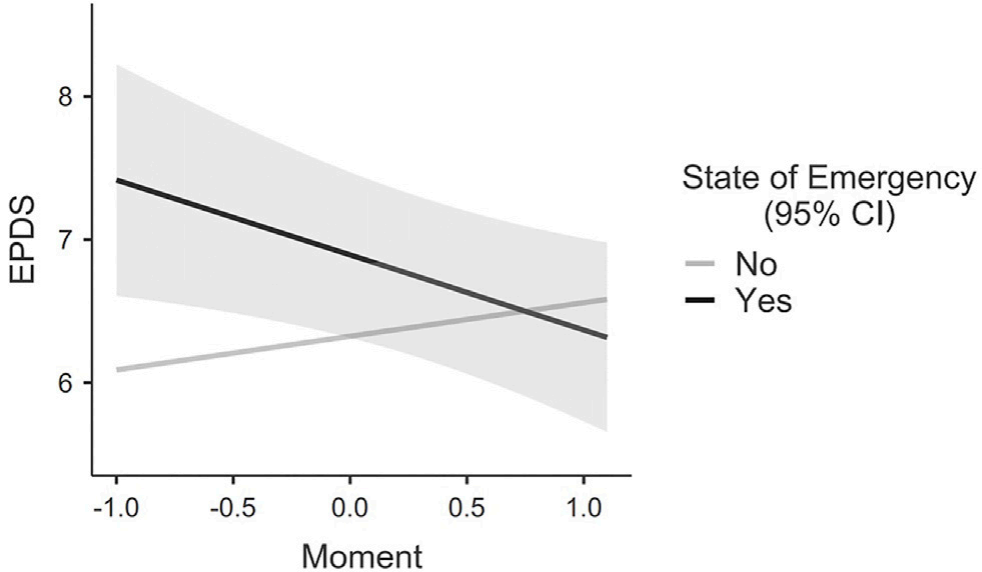
Marginal means interaction plot (Moment*Emergency State).

Similar to Model II, this model also evidenced significant differences in the two random effects (τ00 CI 95% [3.03, 3.83], and τ11 CI 95% [0.08, −0.99]). Looking at the differences in effect τ00 between unconditional and conditional models (II and III), pseudo *R* ^2^ indicated that the state of emergency accounted for 4% of the variance of the EPDS across participants. Additionally, given that the AIC for Model III was lower than that for Model II (where state of emergency was not included), and the chi-square test for deviance was significant (*χ* ^2^ = 13.307, *p* < 0.01), this indicates that the inclusion of the state of emergency led to better model fit. In line with expectations, the fixed effect of the intercept, although continuing to be significant, CI 95% [5.61, 6.62], showed that EPDS scores at T1 when women were not under the state of emergency were lower relative to EPDS scores at T1 when women were under the state of emergency, denoting that the strict COVID-related restrictions had a significant effect in increasing women’s EPDS scores. Interestingly, the model indicated a significant state of emergency effect, CI 95% [0.55, 2.32], with EPDS scores at T1 under the state of emergency being higher by 1.38 points compared to when no restrictions were imposed (state of emergency = 0). The interaction effect Moment*State of Emergency was significant, CI 95% [−1.47, −0.31], indicating a decrease in EPDS scores over time when women were exposed to the restrictions of the state of emergency, and the opposite pattern of increase in EPDS scores when the state of emergency was not decreed (see Figure 1).

As the interaction effect Moment*State of Emergency could be explained by the observed difference in EPDS scores at baseline between the two levels of the variable state of emergency, we computed a final Conditional Growth Model (Model IV), with the interaction EPDS at T1*State of Emergency. The results showed that the Moment*State of Emergency interaction effect of the previous model ceased to be significant, Cl 95% [−0.60, 0.36]. Furthermore, EPDS scores at pregnancy showed significant differences, CI 95% [0.84, 0.92] and the within-subjects variability halved (σ^2^), showing a flat fluctuation. A marginal effect for EPDS at T1*State of Emergency interaction was also noted, Cl 95% [−0.16, 0.00]. Comparing this model (IV) with the previous model (III), we found an increase in *R* ^2^, the AIC, BIC, and reduced −2LL, and the chi-square test for deviance was significant (*χ* ^2^ = 616.85, *p* < 0.0001), showing that this model fitted better than Model III. These results showed that the significant interaction between the state of emergency and the moment, previously significant in model III, was mainly explained by EPDS scores at baseline. The results also seem to show that the value of the EPDS scores at baseline conditioned all the effects of the model, dwindling flutuaction of EPDS over time.

### Relative Importance Analysis

Lastly, we examined the contribution of potential risk and protective factors for women’s depressive symptoms at T1, considering whether they were under strict lockdown measures. This model explained 72% of the variance in EPDS scores at baseline. Relative weights, rescale relative weights, and coefficients directions of each predictor are shown in Table 2.

**TABLE 2:** Relative weight analysis predicting baseline (third-trimester) depressive symptoms using anxiety, dyadic-adjustment, perceived stress, perceived social support, mindfulness, sexual distress, and sexual functioning measured at the same moment (Porto, Portugal, 2021).

Predictors	Global sample		Emergency state YES		Emergency state NO		Group comparison	
	RW	RS-RW	RW	RS-RW	RW	RS-RW	CI. Lower	CI. Upper
HADS	0.26 [+]*	36.81	0.15	22.79	0.29	39.32	**0.032**	0.232*
DASR	0.02 [−]*	3.34	0.03	3.85	0.02	3.24	−0.061	0.035
PSS	0.23 [+]*	31.58	0.20	29.34	0.23	31.35	−0.063	0.127
MSPSS	0.03 [−]*	8.51	0.17	24.24	0.05	6.36	**−0.232**	−0.020*
FFMQ	0.07 [−]*	9.62	0.09	13.46	0.07	8.83	−0.109	0.052
FSDS	0.06 [+]*	8.79	0.03	4.75	0.07	9.60	−0.022	0.097
FSFI	0.01 [−]	1.34	0.01	1.57	0.01	1.29	−0.038	0.030

Note: RW, relative weights, which are scaled in the metric of relative effect sizes (i.e., proportion of variance in the EPDS attributed to the predictor) and sum to the model *R* ^2^; [+] Positive value of GLM estimate [−] Negative value of GLM estimate; RS-RW, rescaled relative weights, which represent the percentage of the predicted criterion space (*R* ^2^) that is attributed to each predictor variable and (within rounding error) sum to 100. *p < 0.05. Bolded values indicate significant effects.

All variables with the exception of sexual functioning were significant predictors of T1 EPDS scores. Together, perceived stress and anxiety scores accounted for approximately 68.4% of the predicted variance of EPDS scores. These results denote that perceived stress and anxiety reported at baseline were positively and significantly associated with depressive symptoms at that time-point. Considering the results per group, social support and anxiety were significant predictors of EPDS scores, indicating that women differed significantly depending on whether they were under the strict lockdown measures of the state of emergency. In fact, anxiety scores explained significantly less percentage of the EPDS variance when women were under strict lockdown, comparing to when women were not under strict lockdown. On the other hand, perceived social support showed the opposite effect, as it had a significantly greater predictive power of depressive symptoms when women were in a state of emergency compared to when they were not. This result shows the impact of social support as a protective factor against depression during strict lockdown measures.

## Discussion

The current pandemic has increased research and clinical demands to understand the wide-reaching impacts on populations’ physical and mental health across the globe. As a result of the pandemic, pregnant and postpartum women have faced disruptive changes to their pre- and post-natal experiences [42, 43], which may have increased the stressful character of this period and make them a particularly vulnerable population. Although Portugal was one of the European governments to implement the strictest and longest confinement and stay-at-home measures, to our knowledge there are currently no longitudinal reports on the impact of the pandemic on the perinatal mental health of Portuguese women. The current study aimed to fill this gap. Across three assessment waves, a sample of Portuguese women who were having their first child during the pandemic was assessed from pregnancy to 6-month postpartum, with the aim of examining the effects of the pandemic on their risk of developing depressive symptoms. Specifically, we assessed whether pandemic-related confinement measures were associated with a heightened risk of developing depressive symptoms across this period, and whether recognised risk and protective factors might heighten or, conversely, protect women against deleterious effects of the pandemic.

Findings add to our understanding of the impact of the pandemic on women’s perinatal mental health by demonstrating evidence of differential vulnerability across specific moments of the transition and of the contribution of targettable risk and protective factors for these women. Specifically, we found that the risk of reporting depressive symptoms was higher when women were in the third trimester and approaching the end of their pregnancy than when women were already at postpartum. Although it is recognised that postpartum is typically a highly demanding period for women—who need to adapt to the novel biological (e.g., physical recovery from childbirth and breastfeeding), psychological (e.g., depression, fatigue), and social (e.g., change in identity and roles) changes of the transition—current findings suggest that the pandemic context may have posed highly demanding stressors to an earlier period typically less vulnerable. The imminence of childbirth while managing the rapidly changing medical guidelines and the perceived lack of social support resulting from the pandemic-related isolation measures (e.g., from the medical staff, from their partners, from family and friends), may have contributed to this increase in depressive symptoms during pregnancy.

Because of the mandatory and rigid physical isolation and social distancing measures that were put in place during the current pandemic, we examined whether being under a state of emergency at the time of assessment could explain the likelihood of reporting greater depressive symptoms. For pregnant women, depressive symptoms were significantly higher by 1.38 points (EPDS score) for those who were under lockdown compared to those who were not under lockdown. This finding supports the idea that strict COVID-related lockdown measures were significantly associated with an increase in women’s depressive symptomatology and it is in line with prior research indicating that, while effective in preventing the spread of the disease, isolation measures also have harmful direct and indirect effects on the physical and mental health of the populations, including negative effects on individuals’ quality of life, interpersonal relationships and, importantly, psychological and physical health [10, 44, 45].

When examining whether there was an added vulnerability of being under strict lockdown at different timings of pregnancy and postpartum (i.e., third pregnancy trimester, 3-, or 6-month postpartum), we found a significant interaction such that depressive symptoms decreased over time when women were exposed to the restrictions of the state of emergency at pregnancy, and the opposite pattern of increase in depressive symptoms over time when women were not under strict lockdown at pregnancy. However, this interaction effect ceased to be significant when the heightened increase in depressive scores at baseline for women who were under lockdown was taken into account. These results indicate that depressive symptoms experienced by women during their third trimester were influential of all subsequent variations in depressive symptoms over time, up to 6-month after childbirth. This confirms prior studies indicating that prenatal depressive symptoms are among the strongest predictors of postnatal depressive symptoms [5] and highlight the importance of targeting pregnant women during pregnancy in order to prevent heightened depressive symptomatology at postpartum, which is of particular importance during the current pandemic.

Building upon models of vulnerability to psychopathology, we examined the role of several recognised protective and risk factors for the development of depressive symptoms during the perinatal period, while considering the experience of lockdown. Since the third trimester of pregnancy was the most vulnerable period for women’s experience of depressive symptoms, as priorly described, we specifically examined the contribution of individual (i.e., anxiety, perceived stress, mindfulness) and relational (i.e., perceived social support, dyadic adjustment, sexual wellbeing) factors to the likelihood of experiencing depressive symptoms between women who were pregnant during lockdown and a control group of women who were pregnant in a period when lockdown measures were not in place. The examined factors accounted for 72% of the variance in women’s depressive symptomatology and, as expected, we found support for the contribution of individual vulnerability factors in both groups. Together, perceived stress and anxiety scores explained 68% of the variance for both groups of women. These results highlight the marked interdependence between perceived stress, anxiety, and depression, aligning with research conducted before and during the current pandemic [5, 12].

Interestingly, when considering lockdown status, anxiety and social support demonstrated contrasting effects on the likelihood of experiencing depressive symptoms. For women who were under strict lockdown, the contribution of their anxiety levels to explain their depressive symptomatology was significantly lower (23%) compared to women who were not under strict lockdown (39%), to whom this contribution was more important. Although the presence of prenatal anxiety is recognised to increase the likelihood of developing maternal illness and that this association can be exacerbated by the current pandemic [12], current findings show that the strength of this link can be different between before and during strict lockdown restrictions. This difference can be explained by the cause of anxiety or other factors that may be more critical during the pandemic in developing depression. Conversely, perceived social support demonstrated the opposite effect, as it had a significantly greater predictive power of depressive symptoms (24%) when women were under strict lockdown compared to when they were not (6%). This emphasises the central role of social support as a protective factor against the development of depression during challenging periods. Indeed, maintaining positive relations of support during pregnancy can help women deal with stress, decrease the likelihood of mothers experiencing postpartum depression, and is directly and indirectly linked to indices of physical and mental health [46–48].

This study contributes to a much-needed area of research during the current pandemic by exploring the intricate and longitudinal associations between external maternal stressors of the COVID-19 pandemic and individual vulnerability factors in the potential development of maternal depression. By employing a longitudinal approach, we were able to explore the temporal associations among these factors, in a particularly vulnerable population as are pregnant and postpartum women. However, current findings should be considered in light of some limitations. Data were collected online, which limited participation to women with access to online resources, but using interviews would be less suitable in the context of a pandemic, possibly increasing noncompliance. We targeted a relatively large sample of women across a period which is typically difficult to monitor, with a good retention rate. It is possible, however, that those participants who dropped out might have presented particular characteristics that prevented them from participating in an online study at this moment of their lives (e.g., lower Portuguese literacy, lower educational background and income level, higher depression). Additionally, all women in this study were in intimate, mixed-sex relationships, were transitioning to parenthood for the first-time, and were healthy at entry. It is unknown whether results generalise to more diverse samples or to those who are faced with additional stressors (e.g., same-sex couples, mothers to an infant born preterm, with comobidities) and this might be explored in future research. In the current study, significant variance was found for women’s depressive symptoms at baseline and over time, with significant variability in trajectories. As such, a group-based approach might prove helpful for future studies to determine specific longitudinal trajectory and predictors of depressive symptomatology during the COVID-19 pandemic.

Perinatal depressive symptoms are among the most common maternal mental health complaints, with a marked proportion of women being at risk for developing postpartum depression [2–4]. The current findings can guide researchers and clinicians in targeting the specific challenges which have emerged during the pandemic for these women, and to develop effective strategies to promote new mothers’ psychological wellbeing.

## Data Availability

The data that support the findings of this study, as well as syntax for all analyses, are available upon reasonable request from the corresponding author, IT.

## Supplementary Material

Supplementary Figure 1 Trelis Plot.
