# Supplementary figures and images for: A Longitudinal Study on Maternal Depressive Symptoms During the COVID-19 Pandemic: The Role of Strict Lockdown Measures and Social Support
Source: Int J Public Health. 2022 Mar 14;67:1604608. doi: 10.3389/ijph.2022.1604608 (PMC8959081; doi:10.3389/ijph.2022.1604608)

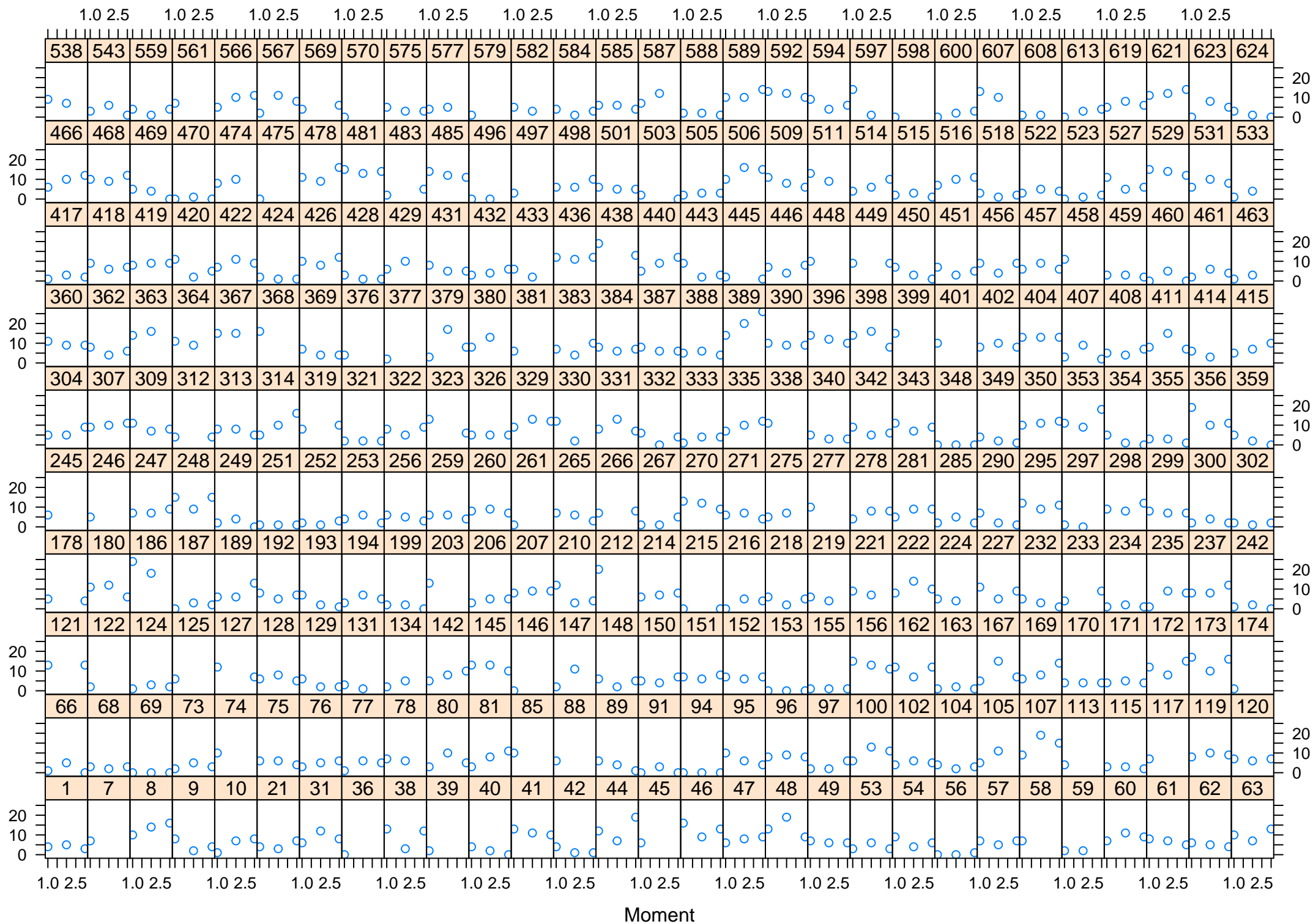

Supplement: Supplementary file 1 [file Image1.pdf]
